# Hopes and Hurdles of Employing Mesenchymal Stromal Cells in the Treatment of Cardiac Fibrosis

**DOI:** 10.3390/ijms222313000

**Published:** 2021-11-30

**Authors:** Sebastian Neuber, Maximilian Y. Emmert, Timo Z. Nazari-Shafti

**Affiliations:** 1 Cardiosurgical Research Group, Department of Cardiothoracic and Vascular Surgery, German Heart Center Berlin, 13353 Berlin, Germany; neuber@dhzb.de (S.N.); emmert@dhzb.de (M.Y.E.); 2 Translational Cardiovascular Regenerative Technologies Group, BIH Center for Regenerative Therapies, Berlin Institute of Health at Charité—Universitätsmedizin Berlin, 13353 Berlin, Germany; 3 Institute for Regenerative Medicine, University of Zurich, 8044 Zurich, Switzerland

**Keywords:** cardiac fibrosis, heart failure, mesenchymal stromal cells, therapy, anti-fibrosis

## Abstract

Excessive cardiac fibrosis plays a crucial role in almost all types of heart disease. Generally, cardiac fibrosis is a scarring process triggered in response to stress, injury, or aging and is characterized by the accumulation of activated myofibroblasts that deposit high levels of extracellular matrix proteins in the myocardium. While it is beneficial for cardiac repair in the short term, it can also result in pathological remodeling, tissue stiffening, and cardiac dysfunction, contributing to the progression of heart failure, arrhythmia, and sudden cardiac death. Despite its high prevalence, there is a lack of effective and safe therapies that specifically target myofibroblasts to inhibit or even reverse pathological cardiac fibrosis. In the past few decades, cell therapy has been under continuous evaluation as a potential treatment strategy, and several studies have shown that transplantation of mesenchymal stromal cells (MSCs) can reduce cardiac fibrosis and improve heart function. Mechanistically, it is believed that the heart benefits from MSC therapy by stimulating innate anti-fibrotic and regenerative reactions. The mechanisms of action include paracrine signaling and cell-to-cell interactions. In this review, we provide an overview of the anti-fibrotic properties of MSCs and approaches to enhance them and discuss future directions of MSCs for the treatment of cardiac fibrosis.

## 1. Introduction

Cardiac fibrosis accompanies most cardiac pathological conditions and is a critical contributor to the progression of heart failure [1]. It is characterized by a remodeling process of the heart in which activated myofibroblasts produce and secrete high levels of extracellular matrix (ECM) proteins in the myocardium. In acute cardiac injury, ECM deposition is essential to preserve the structural integrity of the heart wall and circumvents ventricular aneurysm and eventual rupture [2]. However, uncontrolled and progressive ECM deposition can lead to increased stiffness of the heart, resulting in decreased ventricular filling (diastolic dysfunction) and ventricular contraction (systolic dysfunction), ultimately contributing to the development of heart failure [3]. These characteristic morphological and functional changes that accompany the transition from a healthy to a failing heart are summarized by the term cardiac remodeling [4]. Unfortunately, there is currently no clinically applied therapy that specifically targets myofibroblasts to prevent or even reverse pathological cardiac remodeling during heart failure.

Heart failure is a growing clinical and economic burden worldwide, and despite advances in pharmacological and device therapies that improve survival and quality of life for patients, curative solutions other than heart transplantation are still not available. Conventional therapies for the treatment of heart failure, such as the application of β-blockers, angiotensin-converting enzyme (ACE) inhibitors, and aldosterone antagonists are beneficial for patient survival. Regulation of myofibroblast activity, however, is not the primary target of these pharmaceuticals, but appears to be an additional pleiotropic benefit [5,6]. For example, ACE inhibitors interfere with the renin-angiotensin-aldosterone system by blocking the conversion of inactive angiotensin I into active angiotensin II (Ang II), a potent activator of myofibroblasts [7,8]. When medical therapies are exhausted in the final stage of heart failure, the only therapeutic options that remain are permanent mechanical circulatory support and heart transplantation. However, both have their limitations, and there is still a large discrepancy between the availability of organ donors and recipients [9,10]. Consequently, due to the high morbidity and mortality rate of patients with heart failure, novel therapeutic strategies are urgently needed.

Over the past two decades, cell-based therapy using various cell sources has been extensively studied as a therapeutic strategy for cardiac repair. Most of the cells utilized for this purpose were of mesenchymal origin, ranging from skeletal muscle satellite cells to mesenchymal stromal cells (MSCs), and cardiac progenitor cells (CPCs). More recently, induced pluripotent stem cell-derived progenitor cells have also been investigated as a potential source for the regeneration of injured myocardium [11]. All of these cell therapies have in common that during preclinical evaluation in rodent models of myocardial injury, therapeutic outcome measures, such as angiogenesis, reduction of cardiac fibrosis, and global myocardial function, were improved. However, after application to higher vertebrates such as pigs, the therapeutic effect became less prominent, and most clinical trials with these cells did not provide convincing evidence of myocardial regeneration after injury [12]. Until now, MSCs were the most extensively studied candidates for cell therapy [13], with evidence that the administration of MSCs can improve cardiac remodeling in preclinical models of myocardial infarction (MI) [14,15,16]. MSCs are non-hematopoietic, multipotent, and self-renewing cells that can differentiate into multiple mesenchymal lineages [17,18]. Despite claims demonstrating the expression of cardiac genes on stimulation in vitro, it is well established that MSCs do not differentiate into functional cardiomyocytes in vitro or in vivo [19,20]. Instead, the mechanisms underlying their beneficial effects in vivo are rather related to their paracrine activity [21,22,23,24]. MSCs have the ability to secrete various bioactive molecules, including cytokines, chemokines, growth factors, microRNAs (miRs), and extracellular vesicles (EVs), that have positive effects on failing hearts by attenuating the degree of cardiac fibrosis [25].

In this article, we summarize the anti-fibrotic characteristics of MSCs and ways to stimulate them and discuss the challenges and opportunities of using MSCs to treat cardiac fibrosis.

## 2. Cardiac Fibrosis

Based on recent nuclei data, the adult human heart consists of approximately 20% cardiac fibroblasts (ventricular regions: 15.5%, atrial tissues: 24.3%) [26]. In the healthy heart, cardiac fibroblasts regulate ECM homeostasis through two distinct mechanisms: (i) the synthesis and secretion of ECM molecules and (ii) the secretion of ECM-degrading matrix metalloproteinases (MMPs) and their endogenous inhibitors, so-called tissue inhibitors of metalloproteinases (TIMPs) [27]. The cardiac ECM provides a structural framework for cardiomyocytes and contributes to the mechanical properties and functions of cardiac tissue [28]. However, when resident cardiac fibroblasts are exposed to pressure or volume overload or other pathological stimuli, they become activated and differentiate into myofibroblasts that drive cardiac fibrosis by depositing high levels of ECM proteins [29,30,31].

Cardiac fibrosis is generally categorized into two morphologically distinct forms: reparative and reactive fibrosis [32]. In reparative fibrosis, the death of cardiomyocytes is the key element in stimulating fibrosis; whereas in reactive fibrosis, the death of cardiomyocytes is usually the consequence of fibrosis [33]. Reparative fibrosis occurs in response to injurious stimuli causing cardiomyocyte death, such as MI. Dead cells are replaced by fibrous scar tissue produced by myofibroblasts, which maintains the structural integrity of the ventricles and prevents heart rupture during repair and regeneration but does not replace the function of lost cardiomyocytes [34,35]. In contrast, reactive fibrosis is characterized by excessive deposition of ECM proteins by activated myofibroblasts in the interstitial or perivascular space. It is triggered by stimuli such as mechanical stress due to pressure or volume overload, myocardial inflammation, and metabolic dysregulation related to aging, obesity, and diabetes [36,37,38]. The initial reactive fibrosis occurs as an adaptive response aimed at normalizing increased wall stress and maintaining cardiac output [39]. Excessive reactive fibrosis in interstitial spaces, however, can cause mechanical stiffness and impairment of electric conduction by forming a barrier between cardiomyocytes [40]. Progressive reactive fibrosis in perivascular areas can lead to the narrowing of the vessel lumen, reducing the supply of oxygen and essential nutrients to the myocardium, thereby predisposing cardiomyocytes to ischemic cell death [41]. It is believed that cardiac fibrosis, as a consequence of ischemic heart injury, occurs not only in the immediate infarct area, but possibly also in the infarct border zone and remote cardiac areas. In detail, mouse studies have shown that remodeling processes occur in both infarcted and non-infarcted regions of the heart after an acute MI [42,43].

Multiple cell types are involved in cardiac fibrotic responses, either directly through the production of fibrous tissue (myofibroblasts) or indirectly through the secretion of pro-fibrotic factors (macrophages, mast cells, lymphocytes, cardiomyocytes, and vascular cells) [36]. Pro-inflammatory cytokines (e.g., tumor necrosis factor-alpha (TNF-α), interleukin (IL)-1, and IL-6), chemokines (e.g., monocyte chemoattractant protein-1), and reactive oxygen species may be more important in reparative fibrosis, while mechanical stress, pro-fibrotic growth factors (e.g., transforming growth factor-beta (TGF-β), connective tissue growth factor (CTGF), and fibroblast growth factor-2 (FGF-2)), and hormones (e.g., Ang II) are involved in both reparative and reactive fibrosis [44,45]. Among all these factors, TGF-β is probably the most important primary mediator of cardiac fibrosis and a key mediator in regulating a variety of events during infarct healing [46]. For example, it plays a crucial role in orchestrating the post-infarction inflammatory response, in expanding the myofibroblast population in the healing infarct, and in stimulating the expression of ECM proteins [47,48,49]. TGF-β signaling involves its binding to TGF-β receptor type II, which leads to the recruitment of TGFβ receptor type I (TGFβRI). In the canonical pathway, SMAD2/3 are activated by TGFβRI-mediated phosphorylation, followed by complex formation with SMAD4 and subsequent translocation into the nucleus, where transcriptional reprogramming, relevant to myofibroblast formation, is carried out. In the non-canonical pathway, TGF-β activates SMAD-independent pathways involving, for example, mitogen-activated protein kinases, Rho-like GTPases, and phosphatidylinositol-3-kinase [50]. Finally, in response to pro-fibrotic stimuli, resident cardiac fibroblasts become activated and proliferate and differentiate into myofibroblasts [36]. Phenotypically, compared to cardiac fibroblasts, myofibroblasts express high levels of alpha-smooth muscle actin (α-SMA), ECM proteins, including collagen type I and III, and fibronectin, and have active proliferative, migratory, and secretory properties [51]. Although the expression of α-SMA stress fibers gives these cells the ability to contract, it also creates tension, which, in turn, increases the rigidity of the myocardium [52]. Besides ECM deposition, myofibroblasts also secrete a variety of ECM-degrading proteases, such as MMPs [53,54]. Their immediate function is to disrupt the ECM microstructure so that inflammatory cells can infiltrate local tissue and release additional pro-fibrotic mediators. Myofibroblasts also secrete TIMPs, which reversibly inhibit the activity of MMPs, thereby reducing ECM degradation [55]. By regulating assembly and turnover, myofibroblasts are thus important contributors to ECM homeostasis. Once activated, myofibroblasts also have the ability to produce TGF-β de novo, which acts in an autocrine fashion to induce the proliferation of cardiac fibroblasts and their differentiation into myofibroblasts, ultimately leading to self-persistence of the myofibroblast phenotype [56]. Towards the end of cardiac remodeling, myofibroblasts exit the autocrine loop, likely due to a lessening of pro-fibrotic factors, and may undergo two possible fates: apoptosis or reversible differentiation [57]. In the case of extensive heart damage or comorbid conditions, however, myofibroblasts retain their pro-fibrotic state and actively participate in the pathological remodeling process and the progressive decline in cardiac function.

In sum, while cardiac fibrosis plays an important role in the immediate response to pressure/volume overload or cardiac injury, such as MI, the persistent activation of cardiac fibroblasts can lead to increased heart stiffness and consequent cardiac dysfunction, ultimately resulting in heart failure. Therefore, regulating cardiac fibrosis at the right time and duration is crucial for maintaining and restoring cardiovascular homeostasis. In this context, a previous study showed that early neutralization of TGF-β signaling after MI is harmful as it increases both cardiac dysfunction and mortality, whereas late inhibition of the TGF-β pathway is protective against cardiac fibrosis and adverse cardiac remodeling [58].

## 3. Anti-Fibrotic Characteristics of MSCs

Therapeutic strategies that regulate myofibroblast activity are promising approaches to inhibit cardiac fibrosis from progressing towards cardiomyopathy and thus preventing heart failure. To date, however, there are no effective and safe therapies available that specifically target myofibroblasts, and current medications and surgical interventions only relieve symptoms of heart failure. For patients with end-stage heart failure, continuous mechanical circulatory support or heart transplantation are the only treatment options. Unfortunately, the first is still associated with serious adverse events, such as bleeding, infection, and dependence on external power supplies, thus limiting the overall average life expectancy to only a couple of years [9,59]. In contrast, heart transplantation has better long-term results with an average transplant survival of more than 10 years. However, organ availability is limited, and many patients die while waiting for an organ [60]. To this end, new treatment options were sought, and cell therapy was proposed as an alternative to classical pharmacological approaches. Among the various cell types available for cell therapy, MSCs appeared to be of particular interest because of their ability to evade recognition by the host immune system, their immunomodulatory, pro-angiogenic and anti-fibrotic properties, and their ease of expansion under in vitro culture conditions [61]. Currently, the most commonly used MSCs in preclinical and clinical trials are derived from bone marrow and adipose tissue [62]. Numerous reports have shown that MSCs can improve cardiac function in animal models of heart disease and MI. In general, their beneficial effects during wound healing have been linked in part to their paracrine activity in various tissues, including the myocardium [63,64]. Upon stimulation by injury-mediated soluble factors, MSCs secrete pro-angiogenic factors (e.g., vascular endothelial growth factor, VEGF), anti-apoptotic factors (e.g., insulin growth factor-1, IGF-1), and anti-inflammatory factors (e.g., IL-10) that contribute to the recovery of cardiac function. For example, VEGF has been shown to promote neovascularization of ischemic myocardium in a rat model of MI [65], IGF-1 has beneficial effects on the survival and proliferation of cardiomyocytes [66], and IL-10 attenuates MI by suppressing the inflammatory response [67]. Several preclinical studies have also demonstrated the anti-fibrotic activity of MSCs, as their transplantation significantly reduces fibrosis in the injured heart, thereby attenuating pathological structural remodeling [68,69,70,71,72,73,74,75,76,77,78,79,80,81]. In addition, despite their small sizes, some clinical studies have indicated that MSC transplantation has a positive impact on cardiac repair in humans. For example, significant reductions in scar tissue were found in the POSEIDON and TAC-HFT trials in which patients with chronic heart failure received intramyocardial injections of MSCs [82,83]. Similarly, a significant decrease in the amount of scar tissue after intramyocardial injection of MSCs was seen in patients with severe ischemic heart failure in the MSC-HF trial, but not in the placebo group [84].

To date, one of the greatest limitations of cell therapy is the low level of cell retention after both intramyocardial and intracoronary application [85]. In all of the major clinical trials performed with MSCs, no additional measures were undertaken to increase cell retention. In addition, preclinical studies have shown that standard intramyocardial injection of cells resulted in negligible cell retention within the first 24 h after administration [86,87,88]. However, myocardial remodeling in general, and cardiac fibrosis in particular, is a process that lasts several weeks post-cardiac injury [89]. Consequently, low cell retention rates mainly affect the anti-fibrotic effects of MSCs, as the process of cardiac fibrosis peaks about a week after the initial inflammation phase during the repair phase of cardiac remodeling [47,90]. The optimization of strategies to increase the engraftment and survival of transplanted cells or harness their secretome for clinical purposes is a prerequisite for the clinical implementation of MSCs as a therapeutic approach for cardiac repair [91].

In general, there are four main strategies to target and reverse cardiac fibrosis: (i) elimination of pro-inflammatory factors and their sources, (ii) reduction of oxidative stress and reactive oxygen species, (iii) inhibition of pro-fibrotic pathways, and (iv) direct degradation of the fibrotic ECM. Results from in vitro studies and preclinical trials have demonstrated the ability of MSCs to act on multiple fibrogenesis parameters simultaneously, and the mechanisms of action include both paracrine signaling and direct intercellular communications [80,92,93,94]. One important role of MSCs in attenuating cardiac fibrosis is the regression of inflammation, as chronic inflammation promotes a pro-fibrotic milieu. In a rat model of MI, transplanted MSCs induced a shift in macrophages from a pro-inflammatory M1 state towards an anti-inflammatory M2 phenotype [95]. In addition, in response to MSC treatment, levels of pro-inflammatory mediators, such as TNF-α, IL-1, and IL-6, were decreased [96]. MSC-mediated reduction of oxidative stress is another strategy for targeting cardiac fibrosis, as the activation and expression of TGF-β in myofibroblasts may be mediated by oxidative stress and reactive oxygen species [97]. Furthermore, MSCs can attenuate cardiac fibrosis directly by secreting anti-fibrotic factors; selected examples are given in the following sentences. Hepatocyte growth factor (HGF) is probably the major component responsible for the anti-fibrotic effects of MSCs [94]. It is a negative regulator of cardiac fibroblast differentiation and counteracts TGF-β expression [98]. Adrenomedullin (ADM) is another anti-fibrotic factor secreted by MSCs, which modulates the growth of myofibroblasts through cyclic adenosine monophosphate-dependent signaling [99,100]. Indeed, ADM infusion has been shown to reduce cardiac fibrosis in an ischemia-reperfusion model in rats [101]. Furthermore, anti-fibrotic milk fat globule-epidermal growth factor 8 was found in the MSC secretome, which suppresses the pathogenesis of TGF-β-induced endothelial-to-mesenchymal transition by regulating the activation of related transcription factors [102]. Recently, Qi et al. emphasized the importance of tumor necrosis factor-stimulated gene-6 in the anti-fibrotic response of MSCs by suppressing TNF-α secretion in activated macrophages [103]. Furthermore, the anti-fibrotic effects of MSCs are also related to their ability to produce MMPs, which can directly degrade ECM proteins and enable MSCs to migrate through the ECM [104]. Following the addition of MSC-conditioned medium to cardiac fibroblasts in vitro, decreased cell viability, α-SMA expression, and collagen secretion of myofibroblasts were observed, which was accompanied by an upregulation in the activity of MMP-2 and -9 [93].

Over the past decade, there has been increasing interest in MSC-derived EVs carrying a variety of molecules that can modulate cardiac fibrosis, with regulatory miRs being of particular interest [105]. By definition, miRs are small, non-coding, regulatory RNAs that generally silence gene expression post-transcriptionally by targeting, for example, messenger RNAs to trigger their degradation or inhibit protein translation. They can have multiple functions, including regulating cell physiology, proliferation, cell differentiation, and apoptosis, but may also be involved in regulating cardiac fibrosis [106]. Intriguingly, MSC-derived EVs expressing miR-19a, miR-22, miR-29, and miR-133 were shown to inhibit cardiac fibrosis during heart regeneration and repair [107]. For example, MSC-EVs from bone marrow-derived MSCs containing miR-22 showed anti-fibrotic properties in a mouse model of MI by targeting methyl CpG binding protein 2 expression [108]. The therapeutic benefits of MSC-derived EVs in cardiac repair and regeneration have been successfully reported in preclinical studies, and as they reflect, at least in part, the functions of their parent cells, EVs, are potential tools for next-generation therapeutics [109].

Although paracrine effects have been shown to be primarily responsible for the anti-fibrotic features of MSCs, treatment with conditioned medium from MSCs in vitro cannot fully restore the state of cell therapy in vivo, which involves complex intercellular activities. For example, Li et al. observed physical contacts and tubular structures between myofibroblasts and MSCs [94]. They also found that the inhibitory effects of MSC-conditioned medium on the viability of myofibroblasts and their expression of α-SMA were stronger in a co-culture system. Therefore, in addition to paracrine signaling, both direct cell-to-cell contact and intercellular communication seem to be important for the high therapeutic efficiency of MSCs for the treatment of cardiac fibrosis.

## 4. Modification of MSCs for an Increased Anti-Fibrotic Response

It is expected that efforts to maximize the anti-fibrotic response of MSCs will greatly enhance their beneficial role in regenerative therapies. In general, there are two main strategies: (i) promoting higher expression and secretion of anti-fibrotic factors and (ii) enhancing survival and retention of MSCs at the target site. The first approach could be achieved by genetically modifying cells to overexpress selected anti-fibrotic cytokines or growth factors. For example, Zhao et al. induced overexpression of HGF in MSCs following administration into a mouse model of MI, thereby improving cardioprotection and reducing fibrosis [110]. In addition, the ratio of MMPs to TIMPs in MSCs can be modulated through gene silencing or overexpression. In order to improve the survival of MSCs in vivo, they could be transfected with cell-derived factor-1α (SDF-1α), Akt, integrin-linked kinase (ILK), or islet-1 (ISL1). In detail, in a rat model of MI, it was shown that transplanted SDF-1α-MSCs showed improved tolerance to hypoxic injury and increased viability in infarcted hearts, thereby attenuating cardiac fibrosis [14]. Treatment with MSCs that overexpress the pro-survival protein Akt, a serine-threonine kinase involved in survival and proliferation of MSCs, resulted in a significant decrease in cardiac fibrosis in a pig model of ischemic injury [111]. Similarly, overexpression of ILK in MSCs led to increased survival and reduced cardiac fibrosis in a rat model of MI [112], and overexpression of the LIM-homeobox transcription factor ISL1 improved the survival of transplanted human MSCs in a murine MI model [113]. In addition to genetic modification, the survival of MSCs at the target site can be improved by pre-transplantation treatment with growth factors, such as insulin-like growth factor 1, FGF-2, bone morphogenetic protein-2, sphingosine 1-phosphate, or haemin, as shown in rodent models of MI [114,115,116]. Accordingly, surviving MSCs showed greater efficiency in promoting heart repair and reducing infarct size. Similarly, pre-treatment of MSCs with the hormone melatonin or the pharmacological compound trimetazidine increased their anti-fibrotic activity due to the improvement in MSC survival compared to untreated cells [117,118]. In addition, there is a high probability that a certain degree of cardiomyogenic differentiation of MSCs by pre-exposure to a cocktail of multiple growth factors and induction factors prior to transplantation will result in higher engraftment efficiency [119]. Indeed, the injection of these so-called cardiopoietic MSCs (cpMSCs) led to therapeutic benefits in a mouse model of chronic ischemic cardiomyopathy [120]. A first report on the safety and efficacy of intramyocardial administration of human cpMSCs in immunocompromised pigs after MI showed promising therapeutic results, including a smaller infarct size compared to controls [121]. The first clinical study with cpMSCs was the C-CURE trial [122] with patients suffering from chronic heart failure who were treated with cpMSCs by transendocardial injection. As a result, they showed improved left ventricular ejection fraction and functional capacity compared to control patients who received standard care. These data led to a larger study, the CHART-1 trial [123], in which the effect of cpMSCs in ischemic heart failure was investigated. After 39 weeks, however, there was no difference in the primary efficacy endpoint between patients who received cpMSCs and control patients with no cell injection. At least the subgroup analysis indicated that a subset of the population with more severe cardiac dilation could benefit from these cells. Besides that, hypoxic preconditioning of MSCs has also been shown to increase their retention and survival capacity at the target site and enhance their paracrine abilities [124,125]. In addition, when grown on three-dimensional (3D) platforms prior to clinical use, MSCs have improved regenerative properties by increasing the production of trophic factors and modifying their immunomodulatory and fibrogenic phenotype [126].

**Figure 1:**
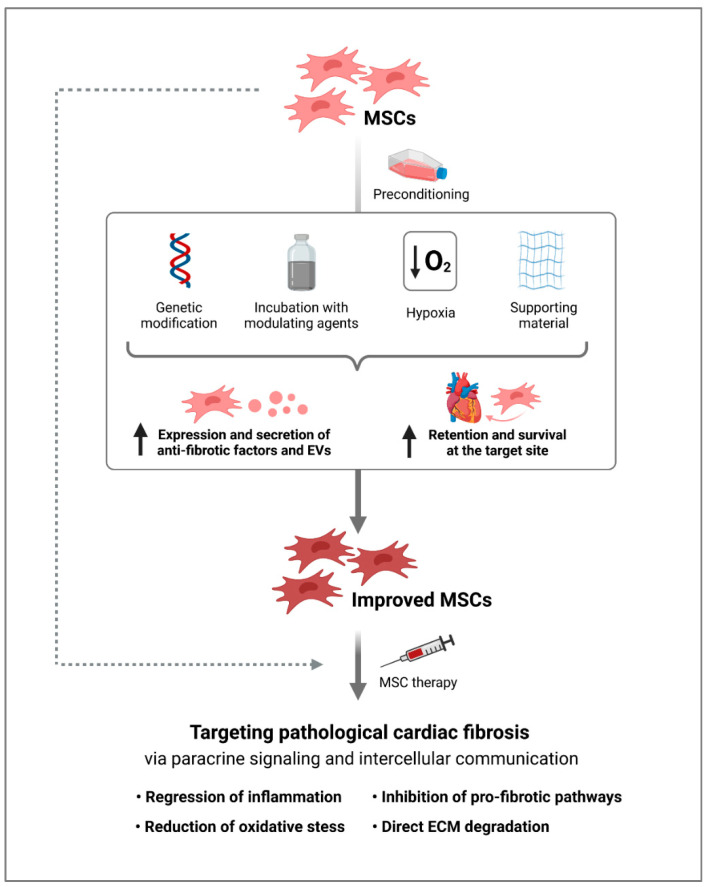
Targeting pathological cardiac fibrosis by MSC therapy. Optimization of MSCs by improving their secretion of anti-fibrotic factors and enhancing their survival and engraftment at the target site through preconditioning prior to transplantation could lead to novel effective MSC therapies for cardiac fibrosis. Intriguingly, as MSC-derived EVs, at least in part, exert therapeutic effects comparable to their parental cells, they represent an alternative cell-free approach for the treatment of cardiac fibrosis (created with BioRender.com, accessed on 29 October 2021). MSCs, mesenchymal stromal cells; EVs, extracellular vesicles.

In summary, various preconditioning strategies for MSCs have been developed to improve their anti-fibrotic properties for an optimized treatment of pathological cardiac fibrosis (Figure 1).

## 5. Challenges and Future Prospects of MSCs for Anti-Fibrotic Therapy

The potential of MSC-based therapies to stimulate cardiac regeneration after MI has been explored for many years, with small animal studies that have shown promising results and large animal trials with encouraging outcomes. However, while endpoints such as scar reduction were observed in large animal trials, functional recovery was not significant even in porcine models of MI [127,128]. Similarly, the majority of clinical trials demonstrated that cell therapy with MSCs did not meet the therapeutic endpoints at a clinically relevant level [129]. Most protocol adjustments regarding administration, cell modification, and timing that could improve MSC performance have only been tested in the preclinical setting. For example, one viable option to increase cell retention is to encapsulate MSCs in the ECM, as shown by Blocki et al. [130]. The aggregation of MSCs into 3D microtissues prior to transplantation is another option to improve cell retention, survival, and engraftment, as demonstrated by a transcatheter-based intramyocardial transplantation of MSC aggregates in a pig model [131,132]. In addition, it has been shown that the self-assembly of cpMSCs into 3D microtissues significantly improved their angiogenic potential and neovascularization capacity [133]. Similarly, for cardiac progenitor cells, Terrovitis and colleagues [86] have shown in animal trials that retention of up to 30% of injected cells for more than three weeks can be achieved when a fibrin-based vehicle is used for administration. The higher retention rate also resulted in a smaller scar size.

Another strategy that has gained traction in recent years is to use EVs from various cell sources to capitalize on the paracrine effects of cell therapy. While there is a large body of preclinical trials in rodent models of myocardial injury utilizing MSC-derived EVs, the majority of porcine trials have been performed with CPC-derived EVs. To date, only Charles and colleagues [134] have utilized MSC-derived EVs to test myocardial recovery after intravenous application of EVs for seven consecutive days post-MI. As a result, they found less cardiac remodeling, indicated by the reduction in infarct size in magnetic resonance imaging, without a pronounced difference in heart function between the intervention and control group. Similar results were achieved with CPC-derived EVs in porcine trials [135,136]. Compared to cell therapy, these cell-free products may also have some advantages from a regulatory point of view. Additionally, EVs can be offered as an off-the-shelf product without the need for expensive cryo storage facilities, which are often needed for cell-based products [137]. Regarding the selection of the cell source for EV production, extensive comparative data is still lacking, but necessary, to determine the most appropriate candidate(s). The advantages and disadvantages of EVs derived from various stem cells for cardiac repair were recently reviewed by Fan et al. [138]. In addition, as a prerequisite for clinical application, further studies on the route, dose, and duration of administration of EVs are necessary, and it must be ensured that MSC-derived EVs can be produced in sufficient quantities and reproducible quality. Furthermore, as there is evidence that MSCs exert their effects through both paracrine effects and direct cell contacts, mimicking intercellular communication should also be considered when developing maximum effective MSC-based products for the treatment of pathological cardiac fibrosis.

## 6. Conclusions

Cardiac fibrotic responses triggered by pathological and environmental stimuli include the recruitment and activation of myofibroblasts, which are critical to physiological cardiac repair in the short term, but these events can also lead to unfavorable scarring and heart failure in the long term. In order to develop new therapeutic strategies, it is important to better understand the processes that either lead to physiological or pathological tissue remodeling. In the last few decades, the application of MSCs has been pursued as a promising approach to mitigate excessive and persistent cardiac fibrosis and to stop the progression towards heart failure due to their paracrine secretion of anti-fibrotic factors. However, the use of MSCs still faces challenges, such as poorly targeted migration, low survival rates at the site of injury, and lack of knowledge about the optimal time and duration of application. Methods for isolating and expanding MSCs, dosage, and cell delivery routes have already been tested in preclinical and clinical trials, but require further investigation. Instead, increasing evidence suggests that EVs derived from MSCs could be an attractive cell-free alternative for reducing pathological cardiac fibrosis. Investigating EVs will provide new insights into the exact mechanism of cardiac regeneration and repair, can help optimize therapies to delay or even prevent the onset of heart failure after an injury, and ultimately reduce the number of people suffering from this disease.
